# Resting Heart Rate Variability, Facets of Rumination and Trait Anxiety: Implications for the Perseverative Cognition Hypothesis

**DOI:** 10.3389/fnhum.2017.00520

**Published:** 2017-10-31

**Authors:** DeWayne P. Williams, Nicole R. Feeling, LaBarron K. Hill, Derek P. Spangler, Julian Koenig, Julian F. Thayer

**Affiliations:** ^1^Department of Psychology, The Ohio State University, Columbus, OH, United States; ^2^Center for the Study of Aging and Human Development, Duke University Medical Center, Durham, NC, United States; ^3^Department of Psychiatry, Duke University Medical Center, Durham, NC, United States; ^4^Section for Translational Psychobiology in Child and Adolescent Psychiatry and the Department of Child and Adolescent Psychiatry in the Centre for Psychosocial Medicine, University of Heidelberg, Heidelberg, Germany

**Keywords:** perseverative cognition, heart rate variability, rumination, anxiety, reflection

## Abstract

The perseverative cognition hypothesis (PCH) posits that perseveration, defined as the repetitive or sustained activation of cognitive representations of a real or imagined stressor, is a primary mechanism linking psychological (or stress) vulnerability with poor health and disease. Resting vagally mediated heart rate variability (vmHRV) is an important indicator of self-regulatory abilities, stress vulnerability and overall health. Those with lower resting vmHRV are more vulnerable to stress, and thus more likely to engage in perseverative cognition and experience subsequent negative mental health outcomes such as anxiety. Recent research suggests that rumination—one of the core mechanisms underlying perseveration—is a construct containing (at least) two maladaptive (depressive and brooding) and one adaptive (reflective) types of rumination. However, to date, research has not examined how the association between resting vmHRV may differ between these three facets of rumination, in addition to these facets’ mechanistic role in linking lower resting vmHRV with greater trait anxiety. The current cross-sectional study explores these relationships in a sample of 203 participants (112 females, 76 ethnic minorities, mean age = 19.43, standard deviation = 1.87). Resting vmHRV was assessed during a 5-min-resting period using an Electrocardiogram (ECG). Both trait rumination (including the three facets) and anxiety were assessed via self-report scales. Significant negative associations were found between resting vmHRV and maladaptive, but not adaptive, forms of perseveration. Similarly, mediation analyses showed a significant indirect relationship between resting vmHRV and anxiety through maladaptive, but not adaptive, facets of rumination. Our findings support the PCH such that those with stress vulnerability, as indexed by lower resting vmHRV, are more likely to engage in maladaptive perseverative cognition and thus experience negative outcomes such as anxiety. Our data also lend a novel outlook on the PCH; resting vmHRV is not related to reflective rumination and thus, this facet of perseveration may be a neutral, but not beneficial, factor in the link between stress vulnerability and psychological well-being.

## Introduction

Perseverative cognition is a common reaction to stressful events and can be defined as the repetitive or sustained activation of the cognitive representations of a real or imagined stressor. The perseverative cognition hypothesis (PCH) proposes that excessive perseveration can have a negative impact on both psychological and physiological well-being, and is often characteristic of those who have difficulties in recognizing signals of safety (Brosschot et al., 2010; Verkuil et al., 2010). Perseverative cognition is thought to be an important mechanism linking psychological or stress vulnerability factors, such as poorer inhibitory control, with psychological outcomes such as burnout, depression and anxiety (Verkuil et al., 2010). Worry and rumination are described as core factors by which perseverative cognition operates. Worry can be defined as the repetitive negative thinking of possible *future* outcomes or events, whereas rumination can be defined as the repetitive thinking of *past* outcomes or events (Nolen-Hoeksema et al., 2008).

### Neurophysiological Concomitants of Perseverative Cognition

While these conceptual definitions of worry and rumination indeed differ, the term perseverative cognition is thought to encompass a common neurophysiological process underlying both constructs (for review, see Verkuil et al., 2010). Specifically, the PCH proposes that the neural concomitants of perseveration involve cortical brain areas associated with appraisal and coping, such as prefrontal (PFC) and anterior cingulate cortices (ACC; Ochsner and Gross, 2008) in addition to subcortical brains areas associated with threat, such as the amygdala (Thayer and Lane, 2002; Anderson et al., 2004; Verkuil et al., 2010). In this regard, it is proposed that engaging in perseveration effectively creates a cognitive representation of the stressor that can maintain a vigilant state, which is thought to be a product of both heightened attention to negative stimuli (via hyperactive amygdala) and a failure to recognize safety within the environment (via hypoactive PFC and ACC; for review, see Verkuil et al., 2010). This framework is not without support, as converging imaging evidence showed decreased activity in the PFC and ACC and increased activity in the amygdala is associated with greater perseveration (e.g., Cooney et al., 2010). Interestingly, this neural circuit has direct neuroanatomical connections to preganglionic sympathetic and parasympathetic neurons (for example, see Barbas et al., 2003; for reviews, see Thayer and Lane, 2000, 2009; for example, see Resstel and Corrêa, 2006). Therefore, bi-directional communication between executive brain areas and the amygdala can be reflected in autonomic nervous system (ANS) activity.

### Resting Vagally Mediated Heart Rate Variability and Perseverative Cognition

The ANS dually innervates many peripheral organs, including the heart, which is under tonic inhibitory control by the parasympathetic nervous system (PNS: a branch of the ANS; for reviews, see Thayer and Sternberg, 2006; Thayer and Lane, 2009; Thayer et al., 2010). The PNS is a critical mechanism in adaptively regulating physiological functions (e.g., inflammation and cardiovascular function) to produce context-appropriate responses via the vagus nerve—the primary nerve of the PNS (Thayer and Sternberg, 2006; Weber et al., 2010). Resting-state^1^ vagally mediated heart rate variability (vmHRV), defined as the rapid beat-to-beat fluctuations in a heart rate (HR) time series, serves as a non-invasive proxy of cardiac vagal control (Task Force of the European Society of Cardiology, 1996; Thayer et al., 2010). As such, resting vmHRV is widely recognized as a psychophysiological index of healthy heart function (Thayer et al., 2010) and overall health (Thayer and Sternberg, 2006; Thayer et al., 2012; Jarczok et al., 2015). Indeed, as resting vmHRV indexes PNS activity (vagal control), resting vmHRV has been linked with activity of executive brain areas, particularly the PFC and ACC. A recent meta-analysis showed that across fMRI investigations, resting vmHRV was positively associated with regional cerebral blood flow in both the PFC and ACC (Thayer et al., 2012), thereby providing neurophysiological evidence of the link between executive brain regions and vagal activity. Behavioral studies also support these notions, having showed resting vmHRV to predict a wide-range of self-regulatory processes, for example emotion regulation (e.g., Williams et al., 2015) and cognitive control (e.g., Anderson et al., 2004; Williams et al., 2016). Therefore, resting vmHRV not only serves as an index of overall health, but also the degree to which the brain’s integrative system for adaptive inhibitory control (e.g., emotion regulation) provides flexible modulation of the periphery. Overall, it is suggested that the aforementioned common neural circuit links psychological processes such as perseveration with health-related physiological processes via the vagus, and that the integrity and flexibility of this circuit can be indexed using resting vmHRV (Thayer et al., 2012).

Taken together, as executive brain areas are responsible for both reducing perseveration and adaptively regulate the vagus nerve, those with lower resting vmHRV may not possess necessary inhibitory abilities to down-regulate perseverative cognition and other negative psychological mindsets. Therefore, the PCH posits that lower resting vmHRV reflects poorer emotion regulation and thus, a greater psychological (i.e., stress) vulnerability—or a greater predisposition—for engaging in perseverative cognition (Thayer and Lane, 2002; Brosschot et al., 2010; Verkuil et al., 2010).

### Resting Vagally-Mediated Heart Rate Variability, Perseveration and Anxiety

As previously mentioned, individuals with higher resting vmHRV typically show better emotion regulation abilities (for review, see Thayer and Lane, 2000; Williams et al., 2015) in comparison to individuals with lower resting vmHRV, and thus may employ better emotion regulation strategies (Appelhans and Luecken, 2006; Volokhov and Demaree, 2010). However, individuals with lower resting vmHRV typically engage in more maladaptive emotion regulation strategies such as perseveration (Brosschot et al., 2010). In this context, perseverative cognition is often thought of as a negative and nonconstructive process; however, recent reports have highlighted that not all perseveration, specifically rumination, is considered maladaptive (for review, see Nolen-Hoeksema et al., 2008). The authors proposed that rumination is a construct containing at least three facets: (i) brooding rumination, defined as the tendency to wallow and sulk over past stressors (maladaptive); (ii) depressive rumination, defined as the tendency to feel sad and despair over past stressors (maladaptive); and (iii) reflective rumination, defined as the tendency to engage in analytical thinking in response to past stressors (adaptive). However to date, research has not yet explored how the relationship may differ between resting vmHRV and these varying facets of perseveration (in this instance, rumination). Likewise, it is well known that rumination plays an important role in anxiety (and depressive) disorders, however little research has reported on the relationship between rumination as a multi-faceted factor and trait anxiety (Nolen-Hoeksema, 2000; for review, see Nolen-Hoeksema et al., 2008).

Moreover, we previously reported that perseverative cognition is an important mechanism linking stress vulnerability (e.g., lower resting vmHRV) with psychological outcomes such as anxiety (for review, see Brosschot et al., 2010; Verkuil et al., 2010) and depression (Stange et al., 2017). A longitudinal report provides evidence in support of this idea, showing that perseveration, specifically rumination, was a significant mediator linking stressful events with both anxiety and depression over the lifespan (Michl et al., 2013). Additionally, a recent fMRI investigation showed that induced perseveration decreased functional connectivity between PFC and amygdala activity in healthy controls—a pattern seen in patients with generalized anxiety disorder in the absence of perseveration (i.e., at rest; Makovac et al., 2016). Importantly, such reductions in connectivity predicted reductions in vmHRV for both groups. Similarly a recent study also showed resting vmHRV to predict the capacity for neural activity to shift from an internally-directed pattern (supporting perseverative cognition) to activity associated with control of externally-directed attention (decreasing perseverative cognition via goal-focused behavior; Ottaviani et al., 2016). Overall, converging neural, physiological and psychological evidence supports the idea that, in comparison to those with higher resting vmHRV, people with lower resting vmHRV have a greater psychological predisposition for perseveration, which can be a maladaptive mechanism linking lower resting vmHRV with negative psychological outcomes, especially anxiety. However to date, no study has investigated the direct relationship among these three variables, especially in light of rumination being described as multi-faceted—including a possible adaptive—factor (Nolen-Hoeksema et al., 2008).

### Present Study

Understanding the strength and direction of the association between resting vmHRV and everyday adaptive and maladaptive perseverative tendencies is warranted due to the paucity of research on reflective rumination and its psychophysiological concomitants. As this form of perseveration—reflective rumination—has the potential to be either non-harmful or beneficial, it is also important to understand the possible mediating impact each facet of rumination may have on the relationship between resting vmHRV and negative psychological outcomes. Thus, the current study sought to explore the direction and strength of the association between resting vmHRV and self-reported ruminative tendencies, including the three aforementioned forms of rumination. We also used mediation models to determine if and how each type of rumination severed as a mechanism linking lower resting vmHRV (an indication of psychological vulnerability for perseverative cognition) with trait anxiety (one possible consequence of lower resting vmHRV coupled with perseverative cognition; Verkuil et al., 2010). If executive brain function is critical in the regulation of both the vagus and perseverative cognition processes, and if the vulnerability of this circuit can be indexed using resting vmHRV, then we hypothesized that lower resting vmHRV would be associated with greater reports of day-to-day perseverative cognition, particularly in both a brooding and depressive (i.e., maladaptive) manner. We also hypothesized that both maladaptive forms of rumination would significantly mediate the association between lower resting vmHRV and greater trait anxiety. We sought to explore the association between resting vmHRV and reflective rumination, in addition to mediating role reflective rumination may (or may not) have on the link between resting vmHRV and anxiety.

## Materials and Methods

Subjects were recruited from the Research Experience Program (REP) pool at The Ohio State University, allowing students to participate in research for partial class credit in an introductory level psychology course. Data were pooled across six studies conducted within our lab. Funding from The Ohio State University College of Social and Behavioral Sciences and College of Arts and Sciences also allowed us to recruit and compensate participants outside of the REP pool resulting in a diverse sample across the university (i.e., students from various majors and cohorts). No individual participated in more than one of the six studies. A total of 203 participants’ (112 females, 76 ethnic minorities, mean age = 19.43, standard deviation = 1.87) data were available for analysis. We asked all participants not to smoke, undergo vigorous physical activity, or drink caffeine 6 h prior to the experiment. Each study was approved by the Ohio State University Institutional review board, and all participants signed written informed consent. A portion of these data has been published elsewhere; however, the focus of those data and results were unrelated to the current investigation (Williams et al., 2015).

In all studies, participants were placed in a soundproof experimental room, equipped with a camera and microphone for safety and instructional reasons, and a high definition TV for stimuli presentation. Participants were given a detailed explanation of the procedures that would take place without indicating the specific hypothesis under the study or manipulations applied. Electrocardiogram (ECG) leads were attached to the subjects and while in a separate control room, the experimenter led the subjects to the initial phases of the experiment. Participants first completed a 5-min baseline period, in which they sat in a resting position with the television displaying a blank, gray screen, and were instructed not to move or fall asleep (spontaneous breathing). Participants either completed an experimental task^2^ followed by a set of self-report questionnaires, or completed a set of self-report questionnaires followed by an experimental task. The total duration for each study was approximately 60 min.

### Vagally Mediated Heart Rate Variability

Cardiac data was recorded continuously throughout each experiment via a 3-lead ECG at a 1000 Hz sampling rate using a Mindware™ 2000D (MW2000D) Impedance Cardiograph package. Electrodes were placed: (1) below the right clavicle; (2) on the left side of the abdomen (below the heart); and (3) on the right side of the abdomen. The variability between successive R-spikes was obtained from ECG recordings to calculate HRV during the baseline. Participants’ successive IBIs, in milliseconds, were extracted using HRV 2.51 Analysis software. IBIs were written in a text file and analyzed using Kubios HRV analysis package 2.0 (Tarvainen et al., 2014), allowing for the calculation of time- and frequency-domain indices of resting vmHRV. Artifacts within the R-to-R series were visually detected, and we applied an artifact correction level that would differentiate and remove artifacts (differing abnormal IBIs from the mean IBI) using a piecewise cubic spline interpolation method. The root mean square of successive differences (RMSSD), measured in milliseconds, was calculated and is considered to be a stable (Li et al., 2009) and valid (Thayer et al., 2010), time-domain measure of vmHRV. Autoregressive estimates were also calculated, yielding high frequency power HRV (HF-HRV, 0.15–0.4 Hz; Thayer et al., 2010). In the present study RMSSD correlated highly with HF power (*r* = 0.90, *p* < 0.001), and thus we only report RMSSD results. Results were identical using HF-HRV (results not shown). Additionally, high-frequency peak values (HF peak) were obtained from the autoregressive analysis as a measure of respiration frequency to control for potential bias (Thayer et al., 2002). RMSSD values were natural log transformed (ln) to fit assumptions of linear analyses.

### Self-Report Questionnaires

Rumination was assessed using the 22-item Ruminative Responses Scale (RRS; Treynor et al., 2003). Participants answered on a scale from 1 (*almost never*) to 4 (*almost always*), (sample item: *How often do you think* about how alone you feel), with higher values representing higher trait rumination (Cronbach’s *α* = 0.922). The RRS contains three subscales used to assess the aforementioned forms of rumination, including: (i) brooding (wallowing and sulking; 5-items; *α* = 0.759); (ii) depressive (sadness and despair; 12-items; *α* = 0.886), and reflective (analytical thinking; 5-items; *α* = 0.773) rumination.

Trait anxiety was assessed using the 20-item Spielberger Trait Anxiety Inventory (STAI-T; Spielberger, 1983). Participants answered on a scale from 1 (*almost never*) to 4 (*almost always*), (sample item: “*I feel pleasant*”). The STAI-T showed excellent internal consistency (Cronbach’s *α* = 0.922).

### Statistical Analyses

All statistical tests were conducted using SPSS (ver. 20, IBM Chicago, IL, USA). Median splits are frequently performed in literature on vmHRV and thus, we performed a median split on lnRMSSD (median value = 3.817) to stratify subjects into high and low resting vmHRV groups to allow for easier comparisons to previous studies. Independent samples *t*-tests were conducted to explore potential differences between groups on all included variables. Zero-order correlation (Pearson’s *r*) tests were used to assess the relationship between lnRMSSD values, RRS scores (including subscales), and STAI-T scores. Partial *r* correlation coefficients were also conducted to assess these relationships while accounting for several important covariates (see “Covariates” section below for details).

A SPSS custom dialog called *PROCESS* (Hayes, 2012) was used to examine how each form of rumination, including total (all forms combined) rumination, may independently mediate the link between resting vmHRV and trait anxiety. In PROCESS, “Model 4” allowed us to specify an independent variable (IV: resting vmHRV), up to four mediating variables (M; total, depressive, reflective and brooding rumination), and a dependent variable (DV; trait anxiety). Relevant covariates were also included in this model (see “Covariates” section below for details). Bootstrapping confidence intervals (CI; 95% interval) with a sampling rate of 5000 (Preacher et al., 2007; see MacKinnon et al., 2004, for details regarding the bootstrapping procedure) were used to determine the significance of each mediating or *indirect* effect. Statistics reported include unstandardized betas (B), standard error (in brackets), and the bootstrapping CI’s (lower limit, upper limit) for each path of the model. CI’s that do not include zero indicate statistical significance. All tests were two-tailed and were analyzed using a set level of significance of *α* = 0.05.

#### Covariates

Ethnicity and sex differences exist in resting vmHRV (Hill et al., 2015; Koenig and Thayer, 2016) and thus, both variables were used as covariates (sex coded as 1 = male, 2 = female; ethnicity coded as 1 = European American, 2 = Other). Other covariates thought to influence vmHRV included respiration (as indexed by HF peak values; Thayer et al., 2002), age (in years; Choi et al., 2006) and BMI (kg/m^2^; Koenig et al., 2014; Williams et al., 2016). To examine potential bias by pooling data across studies, three univariate analysis of variance (ANOVA) tests were conducted to examine differences in RMSSD, rumination, and anxiety across studies. Results showed that there was a significant differences in mean RRS scores across studies only (*F*_(5,197)_ = 2.97, eta = 0.265 *p* = 0.013). Thus, the six studies were given dummy codes (1–6) and was also used as a covariate. In sum, ethnicity, sex, respiration, gender, BMI and experiment number was controlled for in all partial *r* and mediation analyses. In partial *r* correlation tests between rumination subscales and both resting vmHRV and trait anxiety, those coefficients are controlling for the other two respective subscales (i.e., brooding and depressive rumination, in addition to aforementioned covariates).

It is important to note that mediation analyses, in addition to aforementioned covariates, tested the unique variance that each type of rumination contributed to the link between resting vmHRV and trait anxiety (in other words, each test included one form of rumination as a mediator between resting vmHRV and anxiety, while controlling for above mentioned covariates *and* the other two types of rumination).

## Results

Group analyses showed that those in the low resting vmHRV group reported higher trait rumination (including each of the three subtypes) and higher trait anxiety in comparison to the high resting vmHRV group (each *p* < 0.05; see Table 1 for means and standard deviations for both high and low resting vmHRV groups).

**Table 1:** Vagally mediated heart rate variability (vmHRV) group comparisons for variables of interest.

	Range of data (min, max)	High vmHRV	Low vmHRV	*t*	*p*
*n*		101	102		
Age	18, 30	19.50 (1.93)	19.36 (1.82)	−0.540	0.590
BMI	16.54, 47.51	24.06 (4.91)	23.69 (4.91)	−0.542	0.589
Resting vmHRV	2.71, 4.78	4.21 (0.24)	3.45 (0.26)	−21.61	**0.000**
Respiration	0.15, 0.40	0.265 (0.049)	0.268 (0.052)	0.658	0.606
Total rumination	23, 77	42.02 (11.83)	47.75 (12.16)	3.41	**0.001**
Depressive	12, 44	22.11 (6.73)	24.86 (6.42)	2.74	**0.007**
Brooding	5, 20	10.12 (3.17)	11.46 (3.37)	2.92	**0.001**
Reflective	5, 20	9.37 (3.45)	10.33 (3.36)	2.03	**0.044**
Trait Anxiety	22, 74	39.40 (10.29)	44.11 (10.10)	3.29	**0.004**

*Note. This table gives the range of data, mean and standard deviation (in brackets) values on baseline measures. Independent samples t-test statistics include both t and *p* values on the difference between high and low vmHRV groups (significant p values in bold). Age was measured in years; body mass index (BMI) was measured in kg/m^2^; resting vmHRV is represented as the natural log transform of the root mean square of successive differences (lnRMSSD); total rumination represents total score on the Ruminative Reponses Scale (RRS); “Depressive”: depressive rumination subscale of RRS; “Brooding”: brooding rumination subscale of RRS; “Reflective”: reflective rumination of the RRS; Trait Anxiety was indexed using the 20-item Spielberger Trait Anxiety Inventory. Bold values denote statistical significant (*p* < 0.05)*.

**Figure 1:**
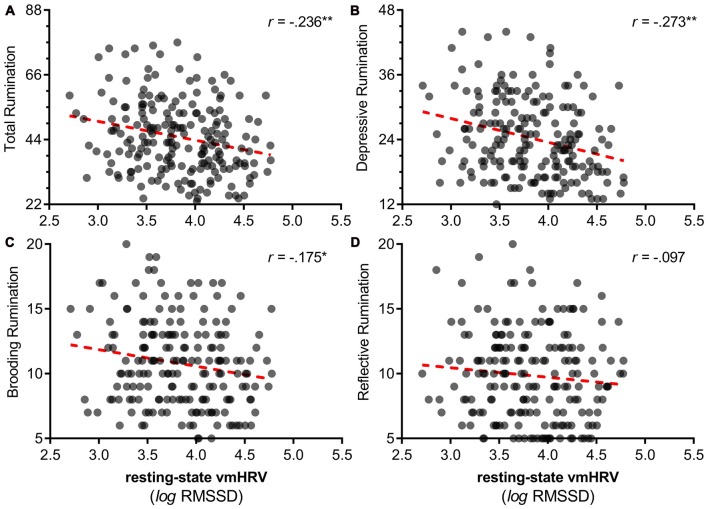
Scatterplots of resting vagally mediated heart rate variability (vmHRV) and different facets of perseveration. *Note*: This figure illustrates scatterplots between resting vmHRV and different facets of perseveration (rumination). **(A)** Resting vmHRV and total (depressive, brooding, reflective) rumination. **(B)** Resting vmHRV and depressive rumination. **(C)** Resting vmHRV and brooding rumination. **(D)** Resting vmHRV and reflective rumination. Resting vmHRV is represented as the natural log transform of the root mean square of successive differences (lnRMSSD). Total rumination represents total score on the Ruminative Reponses Scale (RRS). “Depressive”: depressive rumination subscale of RRS. “Brooding”: brooding rumination subscale of RRS. “Reflective”: reflective rumination of the RRS. *p < 0.05 **p < 0.01.

Zero order correlations showed that lower resting vmHRV was related to higher reports of total rumination (*r* = −0.236, *p* = 0.001; Figure 1A), depressive rumination (*r* = −0.273, *p* < 0.001; Figure 1B), brooding rumination (*r* = −0.175, *p* = 0.013; Figure 1C) and anxiety (*r* = −0.276, *p* < 0.001). Resting vmHRV was not significantly related to reports of reflective rumination (*r* = −0.097, *p* = 0.168; Figure 1D).

Partial *r* correlations controlling for the aforementioned covariates showed a significant inverse relationship resting vmHRV with anxiety (*r*_partial_ = −0.278, *p* < 0.001), total rumination (*r*_partial_ = −0.241, *p* < 0.001), but not brooding (*r*_partial_ = 0.028, *p* = 0.703) or reflective (*r*_partial_ = 0.080, *p* = 0.268) rumination. Results also showed no significant relationship between reflective rumination and trait anxiety (*r*_partial_ = −0.127, *p* = 0.078). Table 2A for zero-order correlation coefficients between all variables, and Table 2B shows partial *r* correlation coefficients between all variables.

**Table 2:** Zero-order and partial *r* correlation matrices.

(A) Zero-order correlation coefficients							(B) Partial correlation coefficients						
	1	2	3	4	5	6		1	2	3	4	5	6
1. vmHRV	-						1. vmHRV	-					
2. Anxiety	−0.276**	-					2. Anxiety	−0.278**	-				
3. Rumination	−0.236**	0.708**	-				3. Rumination	−0.241*	0.720**	-			
4. Depressive	−0.273**	0.723**	0.953**	-			4. Depressive	−0.238*	0.525**	0.950**	-		
5. Brooding	−0.175*	0.643**	0.821**	0.707**	-		5. Brooding	0.028	0.273**	0.825**	0.702**	-	
6. Reflection	−0.097	0.380**	0.768**	0.610**	0.475**	-	6. Reflection	0.080	−0.127	0.760**	0.594**	0.476**	-

Note. Table 2A represents zero-order correlations between variables of interest and Table 2B represents partial correlations controlling for ethnicity, experiment, gender, body mass index, respiration and age. Table 2B represents correlations with additional covariates; in these boxes are correlation coefficients between rumination subscales and both resting vmHRV and trait anxiety controlling for the other two respective subscales (i.e., brooding and depressive rumination). “vmHRV” represents resting vmHRV (the natural log transform of the root mean square of successive differences; lnRMSSD); “Anxiety” represents trait anxiety and was indexed using the 20-item Spielberger Trait Anxiety Inventory; “rumination”: represents total scores on the Ruminative Reponses Scale (RRS); “Depressive”: depressive rumination subscale of RRS; “Brooding”: brooding rumination subscale of RRS; “Reflective”: reflective rumination of the RRS. *p < 0.05 **p < 0.01. Bold values denote statistical significant (p < 0.05).

**Figure 2:**
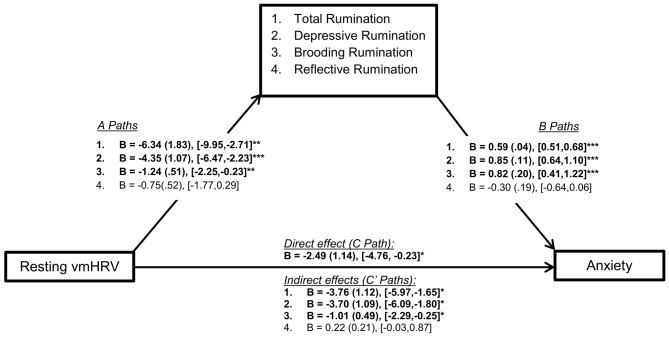
Illustration of mediation model paths and statistics. *Note*: This figure represents the mediation models conducted in the current study. Statistics reported include unstandardized betas (B), standard error (in brackets) and the bootstrapping CI’s (lower limit, upper limit) for each path of the model. CI’s that do not include zero indicate statistical significance. Numbered statistic lines correspond to the respective mediator (facets of rumination). Path A represents the association between the independent variable (resting vmHRV): (1) total (depressive, brooding, reflective) rumination; (2) depressive rumination; (3) brooding rumination; and (4) total rumination. Path B represents the association between these rumination variables and trait anxiety. Path C represents the direct effect between resting vmHRV and anxiety (controlling for all covariates, including total rumination), and Path C’ represents the indirect effect of resting vmHRV on trait anxiety through the varying facets of rumination. Resting vmHRV is represented as the natural log transform of the root mean square of successive differences (lnRMSSD). Total rumination represents total score on the Ruminative Reponses Scale (RRS). “Depressive”: depressive rumination subscale of RRS. “Brooding”: brooding rumination subscale of RRS. “Reflective”: reflective rumination of the RRS. Trait Anxiety was indexed using the 20-item Spielberger Trait Anxiety Inventory. *p < 0.05, **p < 0.01, ***p < 0.001.

Mediation (PROCESS) analyses showed significant mediation (indirect effects) of resting vmHRV on trait anxiety through total rumination (C’: *B* = −3.76 (1.12), [−5.97, −1.65], *p* < 0.05) depressive rumination (C’: *B* = −3.70 (1.09), [−6.09, −1.80], *p* < 0.05), and brooding rumination (C’: *B* = −1.01 (0.49), [−2.29, −0.25], *p* < 0.05). Reflective rumination did not emerge as a significant mediator (C’: *B* = 0.22 (0.21), [−0.03, 0.87]), *p* > 0.05). Specifically, with the exception of reflective rumination, lower resting vmHRV was associated with higher rumination (path A), and higher rumination was associated with higher anxiety (path B). As a result, rumination (total, depressive, brooding) significantly mediated the relationship between resting vmHRV and trait anxiety (significant indirect effect or path C’; see Figure 2 for all path statistics). It is important to note that total, depressive, and brooding rumination only partially mediated the relationship between resting vmHRV and anxiety as the direct effect remained significant despite significant mediation.

## Discussion

The purpose of the current investigation was to explore the possible differential association between resting vmHRV and three facets of rumination, and how each facet may mediate the link between resting vmHRV and trait anxiety. The current results showed that those with lower resting vmHRV reported greater maladaptive perseveration (depressive and brooding rumination). There was no significant relationship found between resting vmHRV and reflective rumination. When examining the association between resting vmHRV and each facet of rumination controlling for other two respective forms of rumination, only depressive rumination was significantly related to resting vmHRV, suggesting that brooding and depressive rumination share similar characteristics as it relates to resting vmHRV. Moreover, both brooding and depressive rumination, but not reflective rumination, mediated (or carried) the relationship between resting vmHRV and trait anxiety, such that lower vmHRV was associated with greater maladaptive perseveration, which was associated with greater trait anxiety. These data lend support for PCH (Brosschot et al., 2010; Verkuil et al., 2010); our data is the first to provide direct evidence that, individuals with lower resting vmHRV report higher maladaptive perseverative cognition and thereby possibly experience negative psychological outcomes such as anxiety.

Importantly, our data also provide a novel outlook on the PCH; although lower resting vmHRV remains a vulnerability trait for maladaptive perseveration, it was not a significant predictor for a more adaptive (Nolen-Hoeksema et al., 2008) form of perseveration, reflective rumination (Path A; see Figure 2). This is further supported by our data, as controlling for aforementioned covariates, including both brooding and depressive rumination, reflective rumination was not a significant predictor of trait anxiety (partial *r* correlation, see Table 2B; path B for reflection rumination, see Figure 2). As a result, reflective rumination did not significantly mediate the relationship between resting vmHRV and anxiety. Although there was no evidence of any benefits of reflective rumination, these null relationships suggest a potentially non-harmful or neutral form of perseveration that is not related to similar stress vulnerabilities (i.e., lower resting vmHRV) that give rise to maladaptive PC.

### Implications

From a neurophysiological perspective, converging evidence links emotion regulation capabilities with executive brain function, such that those with lesser executive brain activity show poorer regulation of negative emotions (Ochsner et al., 2012). During and immediately following a threating or stressful event, amygdala activity is increased, which may be a common and adaptive response given the context specifics. Executive brain regions including the lateral (e.g., Tillfors et al., 2002) and medial (e.g., Sinha et al., 2004) PFC, in addition to the ACC (Drevets and Raichle, 1998), have been implicated in the proper regulation of amygdala activity and any subsequent negative emotions. Therefore when a stressor is no longer present (i.e., under safe conditions), these executive brain regions should exert top-down inhibitory control over the amygdala and other subcortical regions associated with threat and stress (see Wang and Saudino, 2011; for review, Thayer and Lane, 2000, 2002; Verkuil et al., 2010). However, when an individual has reoccurring negative thoughts under safe (stress and threat free) conditions, there can be amygdala activation preceding (worry) or following (rumination) a negative event, coupled with disinhibition (or deactivation) from executive brain regions (Thayer and Lane, 2002; Verkuil et al., 2010). Therefore, perseverative cognition is often characterized as amygdala hyperactivity surrounding a stressful event which is maintained via decreased executive brain inhibition (Thayer and Lane, 2002; Hofmann et al., 2005; Verkuil et al., 2010). It is suggested that a lack of inhibition by executive brain regions does not allow the organism to respond to environmental demands and organize their emotional and behavioral responses adaptively. As such, individuals with lower resting vmHRV under conditions of no apparent threat or stress (i.e., in a safe, resting-state position) are thought to struggle with recognizing signals of safety and thus, are potentially more susceptible to engaging in perseverative cognition (Brosschot et al., 2010; Verkuil et al., 2010). These tendencies can have a negative impact on both psychological (e.g., anxiety) and physiological (e.g., resting vmHRV) well-being and thus, serving as a positive feedback loop that can be detrimental to overall health (Brosschot et al., 2010; see Ottaviani et al., 2016, for meta-analysis; Thayer and Lane, 2002; Verkuil et al., 2010). However, our data do suggest that those who have lower resting vmHRV are not more likely to engage in reflective perseveration as they are to engage in maladaptive perseveration. Therefore, it is possible that the neural concomitants that underlie maladaptive rumination are not completely in line with reflective rumination (Johnson et al., 2009). Further research is needed to fully understand possible neuropsychophysiological mechanisms underpinning reflective rumination, as this may be crucial in understanding how to neutralize the negative impact of maladaptive perseveration (brooding and depressive rumination) on psychological outcomes (i.e., anxiety).

As it relates to stress and disease, this idea of reflective rumination as being a neutral form of perseveration is supported by both the current and prior research. For example, one study found brooding, but not reflective, rumination to mediate the relationship between childhood emotional abuse and depressive symptoms (Raes and Hermans, 2008). Another study found brooding but not reflective rumination to predict the development of depressive symptoms overtime in adolescences (Burwell and Shirk, 2007). In both of these specific examples, reflective rumination showed null results. Likewise, our data showed a null relationship between reflective rumination and both resting vmHRV and trait anxiety, particularly when controlling for other respective rumination facets. In contrast, depressive rumination remained significant under such conditions, and brooding remained significantly related to anxiety but not resting vmHRV. This is important, as taken together, it suggests that the unique variance associated with reflective rumination was unrelated to both resting vmHRV and trait anxiety, possibly generalizing to stress factors and negative outcomes, respectively (Burwell and Shirk, 2007; Raes and Hermans, 2008). In sum, we propose that research examining resting vmHRV and perseverative cognition should consider analytic and reflective forms of perseveration as this may lend further insight on controlling the impact of stress and stress vulnerability on health.

Interestingly, a recent review (Verkuil et al., 2010) on perseverative cognition proposed that perseveration that includes *critical-thinking* and *problem-solving* only further promotes perseveration, and therefore are also associated with negative psychological (and physiological) outcomes (e.g., anxiety). These claims are not without evidence, as those who engage in problem-solving perseveration often find problems unsolvable and over whelming (e.g., Lyubomirsky et al., 1999) and fail to find constructive solutions. In contrast, items of the reflective rumination subscale (e.g., “*Analyze recent events to understand why you are depressed”*) of the RRS are not directed at problem solving and instead, this subscale is thought to reflect the tendency for an individual to *analyze* possible reasons why they are thinking/feeling in a negative manner (i.e., critical thinking) despite being in the presence of safety (for reviews, see Treynor et al., 2003; Nolen-Hoeksema et al., 2008). We propose that this conceptual distinction between adaptive forms of perseveration (i.e., problem solving vs. analytical thinking) serves as an important distinction within the PCH.

### Limitations and Future Directions

One limitation of the current investigation is that the sample consisted of college-aged adults and thus, the current results may not extend to other age ranges. While we are confident that resting vmHRV would be related to perseveration in all age groups, we are not sure of how this relationship may change as a function of reflective rumination. Thus, future research should attempt to examine the link between resting vmHRV and various forms of perseveration in individuals of various age ranges. Other demographic factors, for example socioeconomic status, should be included in future investigations as well.

A second limitation is that the current investigation is cross-sectional and thus, causation cannot be determined. It is plausible to view correlational results as perseveration influencing lower resting vmHRV (i.e., resting vmHRV as DV and perseveration as the IV), as the PCH proposes that resting vmHRV can determine the likelihood of perseveration, which can further impact vmHRV. This positive feedback loop is thought to be a key negative psychophysiological mechanism maintaining the link between stress and disease (see Thayer and Lane, 2002; for reviews, see Brosschot et al., 2010 for empirical study example). However, resting vmHRV has been recently described as an endophenotype (including for anxiety disorders), thus supporting our current conceptualization as an independent variable (for review, see Thayer and Lane, 2009). A recent study lends further evidence in this regard, as it displayed a relationship between parasympathetic inflexibility (i.e., resting-to-reactive vmHRV) and *prospective* symptoms of depression was exacerbated by perseverative cognition (Stange et al., 2017).

Additionally, while bootstrapping techniques in mediation are thought to lend strong support for theorized causal relationships (Hayes, 2012), alternate models for the current study could be proposed. Specifically, models that include resting vmHRV, total rumination only, and anxiety showed significant mediation no matter the variable’s place within the model (i.e., resting vmHRV could have served as an IV, M and DV using these three variables). However, it is important to note that subscales of rumination did not show a similar pattern, as mediation models were only significant with rumination subscales as mediating variables. Moreover, mediation was not significant with trait anxiety as an independent variable, rumination (and three facets) as a mediator, and resting vmHRV as a dependent variable (a switch of the IV and DV in the current study). Thus, differences amongst relationships between resting vmHRV, trait anxiety and *different facets* of rumination are specific to the model as outlined in the current investigation. That is, lower resting vmHRV (independent variable), serves as a psychological predisposition for maladaptive but not adaptive perseveration (mediating variables), which can lead to greater trait anxiety (the dependent variable).

We also acknowledge that the current study is only applicable on a trait, but not state, level; our mediation models cannot determine how individuals may perseverate and experience subsequent consequences from situation to situation. In other words, there may be situations in which individuals may be motivated to ruminate, and thus not experience negative outcomes such as anxiety. Therefore, future work should consider the link between vmHRV, rumination and anxiety as state (situation-to-situation) variables.

A final limitation of the current investigation is that, as resting vmHRV is significantly associated with total rumination scores, some may argue a possibility that non-significant finding between vmHRV and reflective rumination result is a type II error. To explore this issue, we tested if the zero-order correlation coefficients between resting vmHRV and the three subscales significantly differed from one another. The only marginally significant difference found between correlation coefficients was those involving depressive and reflective rumination (*p* = 0.06) such that the correlation between resting vmHRV and depressive rumination was greater than the correlation between resting vmHRV and reflective rumination. No other differences were found. Therefore, despite the significant association between resting vmHRV and total RRS scores, we propose that it is likely due to the unique variance associated with brooding and especially depressive, but not reflective, facets of rumination. Additionally, our median split results showed the high vmHRV group reported significantly lesser reflective rumination compared to the low vmHRV group. Zero-order correlation coefficients also showed a significant positive association between reflective rumination and anxiety. Such patterns of results are inconsistent with our current discussion. However here, we must point out that in paths A and B of the mediation model—when several important covariates are included—only depressive and brooding rumination were significantly related to both resting vmHRV and anxiety. Likewise, partial *r* correlations between resting vmHRV and facets of rumination were controlling for the other respective forms of rumination; resting vmHRV remained related to depressive rumination, and anxiety to both depressive and brooding rumination. However, the associations regarding reflective rumination remained insignificant and small. Taken together, these data suggest that there may indeed be unique characteristics that differentiate reflective and maladaptive, especially depressive, rumination. However as mentioned, future research in needed to better understand each facet of rumination and their unique contributions to stress and disease.

## Conclusion

In sum, our data are the first to provide direct evidence that trait perseveration, specifically self-reported maladaptive rumination (brooding and depressive) are both related to resting vmHRV and can mediate the link between resting vmHRV and self-reported trait anxiety. However, we also find that irrespective of resting vmHRV, individuals are equally likely to report engaging in reflective rumination, and this factor did not mediate the association of resting vmHRV and trait anxiety. Thus, for individuals with stress vulnerability and deficiencies in controlling perseverative cognition processes (e.g., those with lower resting vmHRV), we note that not all forms of perseverative cognition are involved in linking stress vulnerability with the development of anxiety and other negative psychological states.

## Author Contributions

All authors listed have made a substantial, direct and intellectual contribution to the work, and approved it for publication. NRF and DPW are co-first authors; they contributed equally to this manuscript.

## Conflict of Interest Statement

The authors declare that the research was conducted in the absence of any commercial or financial relationships that could be construed as a potential conflict of interest.

## Footnotes

^1^“Resting-state vmHRV” is simply referred to as “resting vmHRV” for the remainder of this report.

^2^Experimental tasks in each study were specific to the primary aims of each investigation.
